# Supplementary material for: Association of Absolute and Relative Handgrip Strength with Prevalent Metabolic Syndrome in Adults: Korea National Health and Nutrition Examination Survey 2014–2018
Source: Int J Environ Res Public Health. 2022 Oct 2;19(19):12585. doi: 10.3390/ijerph191912585 (PMC9564627; doi:10.3390/ijerph191912585)
Supplement: Supplementary file 1 [file ijerph-19-12585-s001.zip › ijerph-1933955-supplementary.pdf]

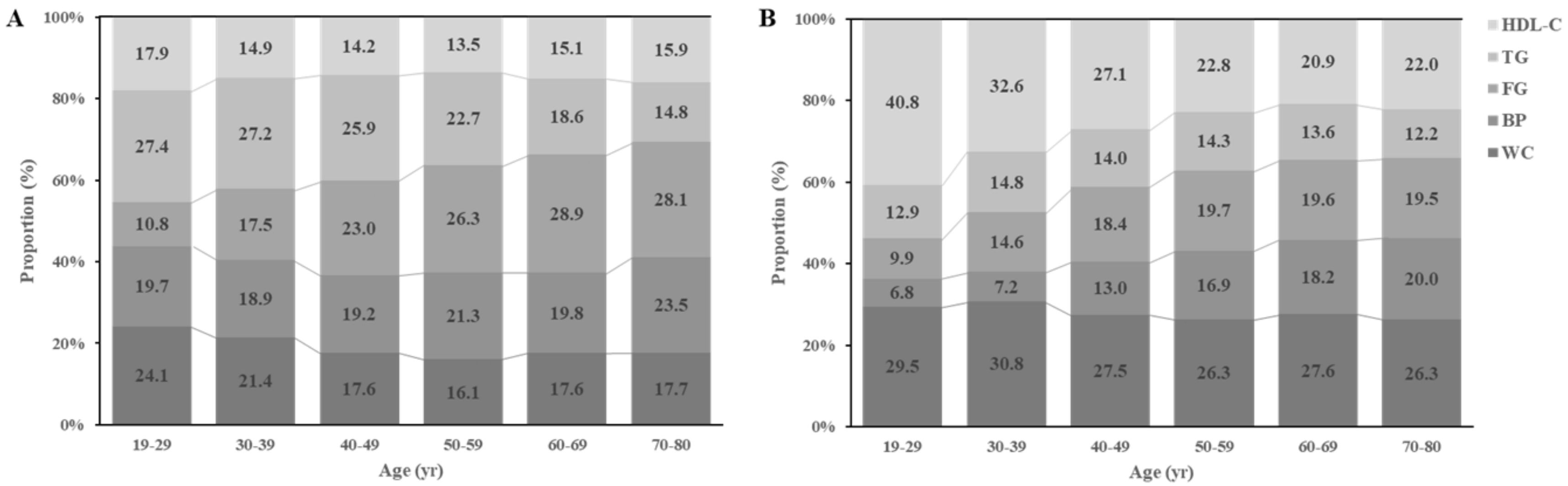

Supplementary Figure S1. Sex-specific pattern of distribution of the MetS components by age groups (A, Males; B, Females)

Abbreviations: HDL-C, high-density lipoprotein-cholesterol; TG, triglyceride; FG, fasting glucose; BP, blood pressure; WC, waist circumference
